# Adaptive Noise Scheduling for Diffusion-based Imputation of Time-series Single-cell Data

**DOI:** 10.1145/3822521.3822557

**Published:** 2026-10-16

**Authors:** Graham Bishop, Gavin Edwards, Haijun Gong, Tong Si

**Affiliations:** Department of Mathematics and Statistics, Saint Louis University, Saint Louis, Missouri, USA; Department of Mathematics and Statistics, Saint Louis University, Saint Louis, Missouri, USA; Department of Mathematics and Statistics, Saint Louis University, Saint Louis, Missouri, USA; Department of Health and Clinical Outcomes Research, Saint Louis University, Saint Louis, Missouri, USA

**Keywords:** Missing value imputation, diffusion model, noise scheduling, single-cell data

## Abstract

Accurate imputation of missing values in single-cell data remains challenging due to high dimensionality and heterogeneous structures, which can not be effectively handled by most traditional imputation methods. Diffusion-based generative models have shown strong performance by learning data distributions through progressive noising and denoising processes. However, these approaches typically rely on fixed noise schedules, and the impact of different noise scheduling strategies on model performance remains insufficiently understood. Our prior work introduced a time-varying blue noise based diffusion model for imputing missing values in time-series data and demonstrated superior performance over many existing methods. Building on this, we systematically investigate a range of noise scheduling strategies and examine how they influence both the noise injection process and overall imputation performance. Experimental results on single-cell RNA sequencing data show that the model is largely insensitive to the ordering of different noise types during injection, and that different noise schedules have limited impact on reconstruction accuracy, thereby providing greater flexibility in the design of noise schedules during model training. These findings offer a promising direction for advancing generative modeling in high-dimensional data analysis.

## 1 Introduction

Time-series single-cell RNA sequencing (scRNA-seq) data are inherently sparse, with a substantial proportion of missing values due to technical limitations [35]. This high level of missingness can severely compromise the accuracy of downstream analyses, including biomarker identification and regulatory network reconstruction. Moreover, most existing network inference methods [13, 22, 32, 33] for single-cell data assume complete observations, making them particularly sensitive to data sparsity. Therefore, developing reliable imputation methods for time-series single-cell data is essential for accurate and robust biological data analysis.

Traditional imputation methods [1, 4, 10] typically assume that missing values follow a known data distribution, which is often unrealistic for high-dimensional time-series scRNA-seq data with complex and heterogeneous structures. Deep generative models [8, 11, 14] have been adopted for modeling high-dimensional data distributions. Among these approaches, the generative adversarial network (GAN)-based methods [19, 20, 23, 36, 37] have shown improved performance over traditional methods. However, these models inherit fundamental limitations of GANs, such as susceptibility to mode collapse and sensitivity to the choice of divergence measures used in the adversarial loss, which can lead to instability during training and reduced robustness in imputation results. Other generative frameworks [7] also exhibit their own limitations when applied to missing value imputation.

To overcome these challenges, diffusion-based generative models [17, 28, 34, 38], built upon denoising diffusion probabilistic models [11], have emerged as a powerful framework for missing data imputation by learning data distributions through progressive noising and denoising processes. However, most existing diffusion-based imputation methods rely on isotropic Gaussian noise, which uniformly perturbs all signal components across diffusion steps. In our recent work, we proposed a time-varying blue noise–based Conditional Score-based Diffusion Model for Imputation [2, 3], demonstrating improved training stability and superior performance on high-dimensional time-series data. Our results suggest that adaptively blending blue and white noise across diffusion stages using a logistic schedule can enhance the noising–denoising process, leading to more effective reconstruction of missing information. Nevertheless, it remains unclear how the ordering of different noise types during injection, as well as the choice of noise scheduling strategy, affect reconstruction accuracy. This motivates a systematic investigation of the role of noise composition and scheduling in influencing both imputation performance and training efficiency in this work.

## 2 Method

Denoising Diffusion Probabilistic Models (DDPMs), originally introduced in [11], provide a generative framework based on a gradual transformation between data and noise distributions. For completeness, we briefly summarize the fundamental components of DDPMs. The framework consists of two complementary processes: a forward diffusion process that incrementally perturbs data with white noise, and a reverse process that learns to recover the original data from noisy observations.

In the forward noising process, the original data is progressively corrupted through a sequence of small Gaussian perturbations. At each diffusion step, the data evolves according to

$$x_{t}=\sqrt{\alpha_{t}}x_{t-1}+\sqrt{1-\alpha_{t}}\varepsilon ,$$

where ε~𝒩(0,I), and $\alpha_{t}\in (0,1)$ controls the noise injection rate at step t. Repeated implementation of this process gradually transforms the data distribution towards an isotropic Gaussian noise. By unrolling the recursion, the forward process can be written in closed form as

(1) $$x_{t}=\sqrt{{\overset{‾}{\alpha }}_{t}}x_{0}+\sqrt{1-{\overset{‾}{\alpha }}_{t}}\varepsilon ,$$

where ${\overset{‾}{\alpha }}_{t}=\prod_{s=1}^{t}\alpha_{s}$, allowing direct sampling of $x_{t}$ from $x_{0}$ without iterating through intermediate steps.

In the reverse process, denoising is carried out sequentially, starting from a noise sample $x_{T}\sim 𝒩(0,I)$, a neural network $\varepsilon_{\theta }\left(x_{t},t\right)$ is trained to estimate the noise component present at each step. This iterative Markovian denoising procedure progressively refines the sample, yielding an estimate of the target data distribution. The reverse transition can be written as

$$x_{t-1}=\frac{1}{\sqrt{\alpha_{t}}}\left(x_{t}-\frac{\beta_{t}}{\sqrt{1-{\overset{‾}{\alpha }}_{t}}}\varepsilon_{\theta }\left(x_{t},t\right)\right)+\sigma_{t}\varepsilon ,$$

where $\beta_{t}=1-\alpha_{t},\sigma_{t}$ controls stochasticity in the reverse step.

Model training can be formulated as a noise prediction task, that is, the network $\varepsilon_{\theta }$ is optimized to recover the noise added during the forward process by minimizing the mean squared error between $\varepsilon_{\theta }$ and ε.

To address missing data, the conditional score-based diffusion model for imputation (CSDI) [28] extends the DDPM framework by explicitly conditioning on observed entries. Let the input $x_{0}$ be decomposed into observed and missing components, denoted by $x_{0}^{\text{obs}}$ and $x_{0}^{\text{mis}}$, respectively. In contrast to standard diffusion models, CSDI restricts noise injection to the missing components only, leaving observed entries intact at all timesteps. Accordingly, in the CSDI model, the Equation 1 is reformulated as:

(2) $$x_{t}^{\text{obs}}=x_{0}^{\text{obs}},\;x_{t}^{\text{mis}}=\sqrt{{\overset{‾}{\alpha }}_{t}}x_{0}^{\text{mis}}+\sqrt{1-{\overset{‾}{\alpha }}_{t}}\varepsilon .$$

The reverse process is formulated as a conditional denoising problem, in which the model learns the distribution of missing components conditioned on the noisy inputs and observed data. The conditional reverse transition is given by

$$p_{\theta }\left(x_{t-1}^{\text{mis}}|x_{t}^{\text{mis}},x_{0}^{\text{obs}}\right).$$

In standard diffusion models, isotropic white noise is injected and removed during the forward and reverse processes, which may fail to preserve frequency-dependent structures present in time-series single cell data. To address this limitation, our recent work [3] introduces a time-varying blue noise–based conditional score-based diffusion model for imputation (tBN-CSDI). Unlike white noise, blue noise exhibits frequency-dependent structure with greater power at higher frequencies, introducing temporal correlations that highlight fine-scale variations while suppressing low-frequency trends. The blue noise $\varepsilon_{\text{blue}}$ has be generated using Ulichney’s void-and-cluster framework [30], combined with simulated annealing and Cholesky decomposition [12] in our method.

In the tBN-CSDI framework [3], we replace the standard Gaussian white noise $\epsilon_{\text{white}}$ used in the original CSDI model with a time-dependent composite noise term ${\tilde{\epsilon }}_{t}$ during the diffusion process. This composite noise is constructed through a smooth interpolation between white noise and blue noise, which is called Blue-to-White (B → W) schedule, defined as

(3) $${\tilde{\epsilon }}_{t}=\gamma_{t}\cdot \epsilon_{\text{white}}+\left(1-\gamma_{t}\right)\cdot \epsilon_{\text{blue}},$$

where the noise blending coefficient $\gamma_{t}\in [0,1]$ controls the contribution of each noise component at diffusion step t, enabling a flexible and time-varying noise scheduling mechanism.

During the forward diffusion stage, the dynamics of the missing components in Eq. 2 are modified by incorporating the composite noise term, yielding

$$x_{t}^{\text{mis}}=\sqrt{{\overset{‾}{\alpha }}_{t}}x_{0}^{\text{mis}}+\sqrt{1-{\overset{‾}{\alpha }}_{t}}\tilde{\epsilon }.$$

In the reverse (imputation) stage, the same composite noise is used for the reconstruction of missing values. Accordingly, the update of the missing components is expressed as

$$x_{t-1}^{\text{mis}}=\frac{1}{\sqrt{\alpha_{t}}}\left(x_{t}^{\text{mis}}-\frac{\beta_{t}}{\sqrt{1-{\overset{‾}{\alpha }}_{t}}}\epsilon_{\theta }\left(x_{t}^{\text{mis}},t|x_{0}^{\text{obs}}\right)\right)+\sigma_{t}\cdot \tilde{\epsilon }.$$

Although our prior tBN-CSDI framework has demonstrated strong performance compared to existing imputation methods, several important questions remain unresolved. The first open question is how the sequence of noise injection strategies influences imputation accuracy and computational efficiency. In our prior work, the early diffusion stages (t≪T) emphasize blue noise, while later stages (t→T) increasingly rely on white noise, which is described by Eq. 3. Alternatively, this schedule can be inverted, with white noise applied in the early stages and gradually transitioning to blue noise, which is called White-to-Blue (W → B) schedule, this process is described by the following equation:

(4) $$\tilde{\epsilon_{t}}=\left(1-\gamma_{t}\right)\cdot \epsilon_{\text{white}}+\gamma_{t}\cdot \epsilon_{\text{blue}}.$$

Another open question is, how different noise blending schedules influence model behavior. The coefficient $\gamma_{t}$ in Eq. 3 and Eq. 4 regulates the blending process by balancing the contributions of the two noise types across diffusion steps. In this work, we examine three blending schedules defined by $\gamma_{t}$, namely step, linear, and logistic/sigmoid functions, which are defined as follows:

**Step schedule:**
$$\gamma_{t}=\left\{\begin{array}{ll} \gamma_{\text{start}}, & t<\tau T,\\ \gamma_{\text{end}}, & t\geq \tau T, \end{array}\right.$$
  where τ∈(0,1) controls the switching point.
**Linear schedule:**
$$\gamma_{t}=\gamma_{\text{start}}+\left(\gamma_{\text{end}}-\gamma_{\text{start}}\right)\frac{t}{T}.$$
**Logistic/sigmoid schedule:**
$$\gamma_{t}=\sigma \left[\gamma_{\text{start}}+\left(\gamma_{\text{end}}-\gamma_{\text{start}}\right){\left(\frac{t}{T}\right)}^{\gamma_{\tau }}\right],$$
  where T is the total number of steps, and $\gamma_{\text{start}},\gamma_{\text{end}}$, and $\gamma_{\tau }>0$ are empirically determined hyperparameters controlling the shape of the schedule. The σ(⋅) is a saturation function (typically a sigmoid) ensuring $\gamma_{t}\in [0,1]$. The logistic/sigmoid schedule was adopted in our previous studies [3].

## 3 Results

In this section, we employ the time-varying blue noise–based conditional score-based diffusion model (tBN-CSDI) for missing data imputation and conduct a series of experiments to systematically investigate the impact of different noise blending strategies and injection order on model performance. We further benchmark our approach against several state-of-the-art imputation methods, such as KNN [1], SoftImpute [10], vanilla CSDI [28], BRITS [5], and GLIMA [27], etc.

Fig. 1 illustrates the three blending schedules using step, linear, and logistic functions considered in our time-varying blue noise-based conditional score-based diffusion model, under different initial values of $\gamma_{t}:0,0.25$, and 0.5.

Our evaluation is based on a time-series scRNA-seq dataset [15], which consists of 120 monocytic THP-1 human myeloid leukemia cells measured at eight time points, yielding a total of 960 cells. Consistent with prior studies [2, 3, 28], imputation performance is evaluated using Root Mean Squared Error (RMSE), Mean Absolute Error (MAE), and Continuous Ranked Probability Score (CRPS) [21], where lower values indicate improved imputation quality. To ensure a reliable evaluation, we simulate missingness by randomly masking observed values at varying rates, following our recent work [3]. Each experiment is conducted over five independent runs, and results are reported as mean performance along with standard errors.

Table 1 presents the imputation performance of the tBN-CSDI model, measured by MAE, RMSE, CRPS, and training time, across three noise blending schedules using step, linear, and logistic sigmoid functions under varying missing rates with $\gamma_{0}=0$ (i.e., no white noise at initialization). Each experiment was repeated five times, and both mean values and standard errors are presented. Overall, the step and logistic schedules exhibit slightly better performance than the linear alternative, although the differences are modest, suggesting that the choice of blending schedule has a limited impact on the overall performance and training time of the proposed tBN-CSDI imputation method.

Our prior work [3] shows that baseline methods, including vanilla CSDI [28], KNN [1], and SoftImpute [10], typically achieve MAE values in the range of 0.6–0.9, RMSE between 0.7 and 1.3, and CRPS around 0.6, with performance degrading as missingness increases. In contrast, The results in Table 1 show that tBN-CSDI consistently outperforms existing methods across all noise blending strategies, achieving MAE of approximately 0.4, RMSE below 0.6, and CRPS around 0.4, corresponding to improvements exceeding 30%. Moreover, the model maintains stable performance across different missing rates and blending schedules, underscoring the effectiveness of blue noise blending for imputing high-missingness scRNA-seq data.

Tables 2 and 3 report the performance of the three noise blending schedules with $\gamma_{0}=0.25$ and 0.5 (i.e., partial white noise at initialization). The results further confirm that tBN-CSDI consistently outperforms traditional methods across all blending strategies. Moreover, its performance remains largely insensitive to both the choice of initial $\gamma_{t}$ and the choice of noise blending schedules, providing greater flexibility in designing noise schedules during model training. This robustness reduces the need for extensive hyperparameter tuning, enhancing the practicality and ease of deployment of the tBN-CSDI model.

Tashiro et al.’s work [28] has showed that vanilla CSDI outperforms several established imputation methods, including V-RIN, BRITS, GLIMA, and RDIS, under various missing rates. Since the results in Tables 1–3 demonstrate that tBN-CSDI consistently outperform vanilla CSDI across different noise blending schedules, it consequently achieves superior performance over all of these competing approaches as well.

Equations 3 and 4 define two noise blending schedules that differ in the order of noise injection during diffusion, including Blue-to-White and White-to-Blue schemes. In our previous work [3], we adopted the Blue-to-White schedule in Eq. 3, where blue noise dominates in early stages and gradually transitions to white noise. In contrast, Eq. 4 adopts the reverse ordering, introducing white noise in the early stages and applying blue noise during later refinement. As shown in Table 4, the step and logistic schedules again exhibit slightly better performance than the linear schedule, consistent with the results reported in Table 1. However, the change in noise injection order has only a minor impact on both imputation accuracy and training time, suggesting that the ordering of noise types plays a limited role in overall model performance. This method not only demonstrates strong performance and robustness on single-cell data, but also generalizes effectively to other large-scale time-series data, for example, healthcare datasets [25].

## 4 Discussion

In this work, we systematically examine how different noise blending schedules, including step, linear, and logistic sigmoid functions, as well as injection orders influence imputation accuracy and training efficiency within the tBN-CSDI framework. Our results show that these design choices have only a minor effect on overall performance. Meanwhile, the time-varying blue noise–based diffusion approach consistently outperforms most state-of-the-art imputation methods across all settings, as the incorporation of blue noise better preserves frequency-dependent structures in the time series data.

Beyond improved accuracy, a key strength of our approach lies in its robustness to both noise scheduling strategies and data sparsity. This stability reduces the need for extensive hyperparameter tuning, enhancing practicality and ease of deployment. Moreover, the flexibility to vary noise blending schedules and injection orders without performance degradation provides a versatile framework adaptable to diverse settings. This is particularly beneficial for heterogeneous, high-dimensional data such as scRNA-seq and clinical time-series, where data characteristics can vary widely.

Although tBN-CSDI demonstrates strong performance, it currently relies on observed experimental time points. In time-series scRNA-seq data, cells measured at the same time point may occupy different biological states, meaning that recorded time does not accurately reflect true biological progression. As a result, imputation based solely on canonical time can overlook important dynamic structure. Pseudotime inference methods [26, 29, 31] address this issue by ordering cells along latent developmental trajectories derived from gene expression profiles. To address this limitation, our recent work will extend tBN-CSDI by incorporating not only experimental time but also pseudotime into the diffusion framework. By aligning the imputation process with inferred cellular trajectories, this approach should better capture biologically meaningful dynamics and improve the reconstruction of gene expression patterns, enabling more accurate modeling of complex cellular processes than the canonical time based methods.

Another important direction for future work is to extend the tBN-CSDI framework to explicitly account for change points in non-stationary time-series single-cell data. Many biological processes, such as cell differentiation and immune responses [9], exhibit stage shifts where gene expression distributions change over time. These transitions are not explicitly modeled in current imputation approaches, which typically assume a uniform noise structure across diffusion steps.

To address this limitation, a promising extension is to incorporate change-point detection methods [6, 16, 18, 24] into the diffusion framework, enabling adaptive noise scheduling and denoising dynamics across different temporal regimes or stages. For example, the noise blending coefficient $\gamma_{t}$ could be conditioned not only on the diffusion step but also on inferred stage or change-point information, allowing the model to apply different noise characteristics before and after change points. This change-point-aware extension would improve robustness to non-stationarity and enhance the recovery of dynamic gene expression patterns. Furthermore, jointly incorporating pseudotime and change-point information into the imputation model provides a unified framework for capturing both continuous progression and discrete transitions, potentially leading to improved imputation accuracy in time-series single-cell data.

## Figures and Tables

**Figure 1:**
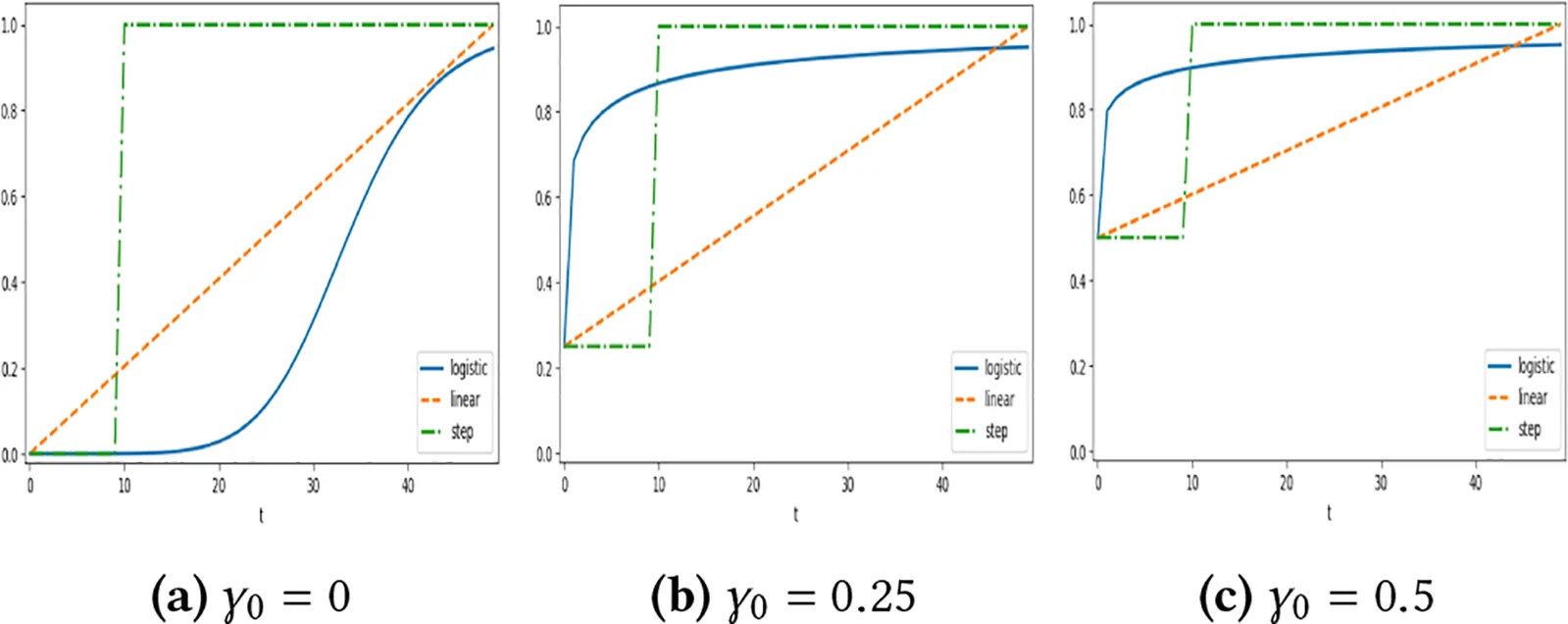
Visualization of three blending schedules in the tBN-CSDI model: step (green), linear (red), and logistic (blue), under different initial values of $\gamma_{t}$.

**Table 1:** Comparison of MAE, RMSE, CRPS, and runtime (in seconds) for the tBN-CSDI method using different noise blending schedules with $\gamma_{0}=0$.

Schedule	Missing Rate	MAE	RMSE	CRPS	Training Time
Step	0.1	0.402 (0.013)	0.590 (0.044)	0.400 (0.011)	54.09 (3.09)
	0.3	0.400 (0.005)	0.583 (0.021)	0.401 (0.005)	52.87 (0.24)
	0.5	0.402 (0.005)	0.567 (0.009)	0.397 (0.006)	52.72 (0.25)
	0.7	0.403 (0.005)	0.569 (0.003)	0.398 (0.005)	53.12 (0.40)
	0.9	0.418 (0.004)	0.591 (0.007)	0.406 (0.001)	53.44 (0.17)
Linear	0.1	0.419 (0.010)	0.608 (0.043)	0.410 (0.009)	54.54 (2.84)
	0.3	0.416 (0.012)	0.646 (0.129)	0.407 (0.005)	53.62 (0.32)
	0.5	0.424 (0.008)	0.600 (0.010)	0.408 (0.003)	54.09 (0.50)
	0.7	0.435 (0.009)	0.611 (0.012)	0.417 (0.007)	54.19 (0.50)
	0.9	0.443 (0.004)	0.632 (0.004)	0.421 (0.002)	54.28 (0.44)
Logistic	0.1	0.403 (0.011)	0.584 (0.032)	0.403 (0.011)	54.77 (2.47)
	0.3	0.404 (0.007)	0.578 (0.011)	0.401 (0.005)	54.18 (0.38)
	0.5	0.408 (0.011)	0.578 (0.013)	0.401 (0.005)	54.36 (0.31)
	0.7	0.420 (0.006)	0.597 (0.009)	0.407 (0.004)	54.45 (0.24)
	0.9	0.433 (0.007)	0.618 (0.011)	0.414 (0.005)	54.24 (0.23)

**Table 2:** Comparison of MAE, RMSE, CRPS, and runtime (in seconds) for the tBN-CSDI method using different noise blending schedules with $\gamma_{0}=0.25$.

Schedule	Missing Rate	MAE	RMSE	CRPS	Training Time
Step	0.1	0.408 (0.016)	0.677 (0.231)	0.407 (0.013)	52.74 (0.64)
	0.3	0.398 (0.008)	0.564 (0.018)	0.400 (0.009)	52.97 (0.42)
	0.5	0.405 (0.009)	0.573 (0.019)	0.401 (0.007)	53.20 (0.33)
	0.7	0.406 (0.005)	0.571 (0.007)	0.401 (0.004)	53.39 (0.14)
	0.9	0.417 (0.008)	0.589 (0.012)	0.405 (0.005)	52.77 (0.18)
Linear	0.1	0.408 (0.014)	0.630 (0.086)	0.398 (0.014)	54.06 (2.42)
	0.3	0.397 (0.007)	0.573 (0.017)	0.392 (0.007)	52.95 (0.30)
	0.5	0.396 (0.004)	0.562 (0.005)	0.391 (0.004)	53.23 (0.21)
	0.7	0.403 (0.009)	0.573 (0.011)	0.396 (0.005)	53.44 (0.13)
	0.9	0.416 (0.004)	0.593 (0.007)	0.401 (0.001)	53.85 (0.30)
Logistic	0.1	0.420 (0.018)	0.729 (0.308)	0.412 (0.011)	53.21 (1.21)
	0.3	0.412 (0.003)	0.610 (0.053)	0.405 (0.006)	53.41 (0.76)
	0.5	0.417 (0.010)	0.593 (0.011)	0.404 (0.005)	53.71 (0.27)
	0.7	0.426 (0.008)	0.606 (0.010)	0.409 (0.005)	53.85 (0.57)
	0.9	0.439 (0.009)	0.630 (0.015)	0.418 (0.007)	53.40 (0.30)

**Table 3:** Comparison of MAE, RMSE, CRPS, and runtime (in seconds) for the tBN-CSDI method using different noise blending schedules with $\gamma_{0}=0.5$.

Schedule	Missing Rate	MAE	RMSE	CRPS	Training Time
Step	0.1	0.403 (0.008)	0.588 (0.048)	0.401 (0.007)	55.30 (3.15)
	0.3	0.395 (0.009)	0.575 (0.011)	0.396 (0.005)	54.60 (0.35)
	0.5	0.402 (0.012)	0.566 (0.009)	0.398 (0.005)	54.58 (0.43)
	0.7	0.405 (0.010)	0.573 (0.008)	0.400 (0.003)	54.94 (0.28)
	0.9	0.415 (0.005)	0.589 (0.005)	0.404 (0.003)	54.88 (0.30)
Linear	0.1	0.406 (0.005)	0.588 (0.029)	0.397 (0.010)	53.16 (0.24)
	0.3	0.393 (0.005)	0.586 (0.045)	0.389 (0.005)	52.89 (0.12)
	0.5	0.397 (0.006)	0.565 (0.013)	0.391 (0.006)	53.06 (0.21)
	0.7	0.405 (0.010)	0.577 (0.015)	0.395 (0.006)	52.96 (0.19)
	0.9	0.413 (0.006)	0.591 (0.008)	0.399 (0.002)	53.05 (0.15)
Logistic	0.1	0.413 (0.015)	0.624 (0.066)	0.404 (0.014)	52.62 (0.33)
	0.3	0.404 (0.005)	0.602 (0.055)	0.398 (0.004)	53.07 (0.26)
	0.5	0.410 (0.006)	0.587 (0.008)	0.400 (0.002)	53.52 (0.49)
	0.7	0.414 (0.008)	0.590 (0.011)	0.401 (0.004)	53.43 (0.25)
	0.9	0.428 (0.005)	0.611 (0.011)	0.411 (0.003)	53.94 (0.28)

**Table 4:** Comparison of MAE, RMSE, CRPS, and runtime (in seconds) for the tBN-CSDI method using reverse noise injection order (White → Blue).

Schedule	Missing Rate	MAE	RMSE	CRPS	Training Time
Step	0.1	0.396 (0.014)	0.557 (0.018)	0.392 (0.008)	56.87 (2.29)
	0.3	0.394 (0.006)	0.559 (0.006)	0.392 (0.005)	55.48 (0.16)
	0.5	0.407 (0.008)	0.576 (0.011)	0.397 (0.001)	55.55 (0.55)
	0.7	0.409 (0.005)	0.579 (0.007)	0.400 (0.003)	56.20 (0.53)
	0.9	0.419 (0.006)	0.600 (0.008)	0.407 (0.003)	56.21 (0.32)
Linear	0.1	0.426 (0.010)	0.599 (0.018)	0.406 (0.015)	56.71 (3.51)
	0.3	0.416 (0.008)	0.592 (0.018)	0.401 (0.008)	56.11 (0.20)
	0.5	0.423 (0.007)	0.603 (0.011)	0.405 (0.006)	56.49 (0.54)
	0.7	0.422 (0.008)	0.601 (0.013)	0.406 (0.006)	56.50 (0.51)
	0.9	0.435 (0.007)	0.624 (0.009)	0.416 (0.004)	56.52 (0.17)
Logistic	0.1	0.392 (0.009)	0.554 (0.016)	0.394 (0.010)	56.67 (0.30)
	0.3	0.391 (0.008)	0.552 (0.009)	0.392 (0.006)	57.01 (0.41)
	0.5	0.394 (0.007)	0.557 (0.005)	0.393 (0.006)	57.54 (0.23)
	0.7	0.403 (0.011)	0.569 (0.014)	0.399 (0.004)	57.56 (0.08)
	0.9	0.412 (0.007)	0.588 (0.010)	0.402 (0.005)	57.88 (0.44)

## CCS Concepts

- Applied Computing → Bioinformatics.
